# Omega-3 Free Fatty Acids Suppress Macrophage Inflammasome Activation by Inhibiting NF-κB Activation and Enhancing Autophagy

**DOI:** 10.1371/journal.pone.0097957

**Published:** 2014-06-09

**Authors:** Yolanda Williams-Bey, Cedric Boularan, Ali Vural, Ning-Na Huang, Il-Young Hwang, Chong Shan-Shi, John H. Kehrl

**Affiliations:** B Cell Molecular Immunology Section, Laboratory of Immunoregulation, National Institute of Allergy and Infectious Diseases, National Institutes of Health, Bethesda, Maryland, United States of America; University of California Merced, United States of America

## Abstract

The omega-3 (ω3) fatty acid docosahexaenoic acid (DHA) can suppress inflammation, specifically IL-1β production through poorly understood molecular mechanisms. Here, we show that DHA reduces macrophage IL-1β production by limiting inflammasome activation. Exposure to DHA reduced IL-1β production by ligands that stimulate the NLRP3, AIM2, and NAIP5/NLRC4 inflammasomes. The inhibition required Free Fatty Acid Receptor (FFAR) 4 (also known as GPR120), a G-protein coupled receptor (GPR) known to bind DHA. The exposure of cells to DHA recruited the adapter protein β-arrestin1/2 to FFAR4, but not to a related lipid receptor. DHA treatment reduced the initial inflammasome priming step by suppressing the nuclear translocation of NF-κB. DHA also reduced IL-1β levels by enhancing autophagy in the cells. As a consequence macrophages derived from mice lacking the essential autophagy protein ATG7 were partially resistant to suppressive effects of DHA. Thus, DHA suppresses inflammasome activation by two distinct mechanisms, inhibiting the initial priming step and by augmenting autophagy, which limits inflammasome activity.

## Introduction

Inflammation is the hallmark of many chronic diseases including type II diabetes, heart disease, cancer, and arthritis. According to the Centers for Disease Control (CDC) chronic diseases account for greater than 50% of all deaths. Understanding the molecular mechanisms that control inflammation and inflammatory pathways is critical to develop new methods to manipulate these pathways and ultimately dampen inflammatory responses in patients. It has been suggested that increasing the intake of ω3 free fatty acid (FFA) by supplementation or by dietary intake of foods rich in ω3 FFA significantly improves the health of patients suffering from chronic inflammatory diseases [1]. A recent report showed that the ω3 fatty acid docosahexaenoic acid (DHA) can inhibit the production of pro-inflammatory cytokines such as TNF-α and IL-6 in RAW 247.6 cells and in primary mouse macrophages by binding to a G-protein coupled receptor (GPR) termed Free Fatty Acid Receptor (FFAR) 4, also known as GPR120 [2].

Inflammasomes serve as central regulators of innate immunity and inflammation [3]. A cytosolic protein complex assembled following the exposure of cells to specific pathogens or “danger” signals inflammasomes contain a nucleotide-binding domain and leucine-rich repeat containing protein (NLR) or Absent in Melanoma 2 (AIM2), which serves as the sensor; an adapter protein ASC (apoptosis-associated speck-like protein containing a caspase activation and recruitment domain); and caspase-1. NLRP1b inflammasomes detect anthrax lethal toxin, the NLRC4 inflammasomes recognize bacterial flagellin, AIM2 inflammasomes are essential for host defense against certain intracellular bacteria and DNA viruses, and a broad range of toxic stimuli trigger the assembly of NLRP3 inflammasomes (4). The activated sensor recruits ASC through homophilic interactions of pyrin domains and ASC associates with pro-caspase-1 via CARD-CARD (caspase activation and recruitment domain) interactions, a step needed to induce caspase-1 activation [4]. The activation of caspase-1 results in the cleavage of IL-1β and IL-18 precursors to their mature forms and their eventual secretion. Several studies have found that the saturated FFA palmitate acid can trigger inflammation by activating inflammasomes [5], [6]. We tested whether the ω3 FFA DHA might have the opposite effect on macrophages and suppress inflammasome activation, thereby reducing IL-1β secretion.

## Materials and Methods

### Ethics Statement

The animal experiments and protocols were performed according to the regulations of the National Institutes of Allergy and Infectious Diseases (NIAID) Animal Care and Use Committee at the National Institutes of Health. The NIAID Animal Care and Use Committee approved this study.

### Animals, THP-1 cells, and bone marrow derived macrophages (BMDMs)

Wild-type C57BL/6 mice were purchased from Jackson laboratory. The *Atg7* ^flox/flox^ mice have been previously described [7] and have been partially back-crossed on to C57BL/6 (3 generations). The mice were kindly provided by Dr. Masaaki Komatsu (Tokyo, Japan) and were crossed with B6.Cg-Tg(Vav1-cre)A2Kio/J mice (Jackson Laboratory) to disrupt the *Atg7* coding region in hematopoietic cells and are referred to as *Atg7* ^flox/flox^ *Vav-1Cre*. The C57BL/6 Green fluorescent protein (GFP)-LC3 (also known as MAP1LC3A) mice were purchased from RIKEN Bio-Resource Center after receiving permission from Dr. N. Mizushima (Tokyo, Japan)[8]. The *arrb1* ^−/−^ and *arrb2* ^−/−^ mice are on a C57BL/6 background and were kindly provided by Dr. Robert J. Lefkowitz (Duke University) [9]. All mice were 6–12 weeks of age at use. Mice were housed under specific pathogen–free conditions. The preparation of mouse bone marrow derived macrophages (BMDMs) and the THP-1 cells have been described previously [10].

### Reagents

The following reagents were purchased: docosahexaenoic acid (DHA) and eicosapentaenoic acid (EPA, Cayman Chemical); Poly(dA:dT), adenosine triphosphate (ATP), and nigericin (Sigma-Aldrich); Lipopolysaccharide (LPS, ENZO Life Sciences); purified flagellin (InvivoGen); neutralizing antibody to IL-1β (R&D Systems); caspase-1 antibody (Santa Cruz Biotechnology); NLRP3 antibody (ENZO Life Sciences); ASC antibody (Santa Cruz Biotechnology); and the appropriate secondary antibodies (Jackson ImmunoResearch Laboratory). For the ImageStream analysis a primary rabbit polyclonal NF-κB/p65 antibody (SantaCruz Biotechnology) was used with an Alexa647 conjugated donkey anti rabbit IgG antibody (Jackson ImmunoResearch Laboratories). DNA was stained using DAPI (20 µM).

### Inflammasome activation

For inducing NLRP3 inflammasome activation, 1×10^6^ macrophages were plated in 12-well plates overnight in complete media (RPMI 1640 plus 10% fetal bovine serum). The following morning the cells were switched to opti-MEM media and LPS (20 ng/ml) or LPS plus various concentrations of DHA were added. DHA was diluted 1∶10 in opti-MEM media from an ethanol stock, vortexed, and added to the cells. Three hrs later ATP (1 mM) or nigericin (1 µM) was added for 1 hr or 2 hrs, respectively. Afterwards, the cells and supernatants were harvested for analysis. For AIM2 inflammasomes, 1×10^6^ macrophages were plated in 12-well plates overnight in complete media and the following morning the cells were primed with LPS (1 µg/ml) for 3 hrs, the cells were washed, transfected with Poly(dA:dT) (0.5 µg/ml) using Lipofectamine (Invitrogen) and supernatants and lysates collected 1 hr later. For NAIP5/NLRC4 inflammasomes, a similar protocol was used except flagellin was transfected using Profect P1 (TargetingSystems).

### siRNA transfection and quantitative RT- PCR

The siRNA pools targeting human *FFAR4* (sc-60737), *GPR84* (sc-60751), or a control (sc-37007, Santa Cruz Biotechnology) were prepared with 2 µl HD Transfection Reagent (Promega) and used at a concentration of 40 nM to treat differentiated THP-1 cells overnight. One day later the cells were primed with LPS and treated with ATP as above. Cells were analyzed from 1 h to 8 h after inflammasome activation. RNA was isolated with TRIZOL Reagent (Invitrogen) according to the manufacturer's instructions. Complementary DNA (cDNA) was synthesized from 1 µg RNA with Omniscript RT Kit (QIAGEN). Real-time PCR was performed using a StepOne™ Real Time PCR System (Applied Biosystems) following the Rotor-Gene SYBR Green PCR kit (QIAGEN) protocol. The following primer pairs were used:

*Ffar1* F:5'-GCTATTCCTGGGGTGTGTGT-3' R:5'-CCCTGTGATGAGTCCCAACT-3'

*Gpr84* F:5'-TCCAATTCTGTCTCCATCCT-3' R:5'-CTGACTGGCTCAGATGAAA-3'

*Ffar4* F:5'-CCATCCCTCTAGTGCTCGTC-3' R:5'-TGCGGAAGAGTCGGTAGTCT-3'

*FFAR1* F:5'-CAGTCTCTCTGCCCCTGAAG-3' R:5'-CGGCATAGAGTGGGAAGAAG-3'

*GPR84* F:5'-TCAGCAGTGTTGGCATCTTC-3' R:5'-CTTGCCTGTCGCAACTTGTA-3'

*FFAR4* F:5'-CCTGAGGTCAGGAGTTCGAG-3' R:5'-CACCACCACTCCCAGCTAAT-3'.

The results were normalized to the expression levels of the β-actin mRNA and the relative mRNA levels were calculated using the 2^−ΔΔCt^ method.

### Immunoblot analysis, confocal microscopy, and Bioluminescence resonance energy transfer (BRET) analysis

Immunoblotting and immunoprecipitations were performed as previously described [10]. For confocal imaging the cells were fixed in cold methanol, immunostained, and imaged with a TCS-SP5 X Supercontinuum confocal microscope equipped with an argon-white laser (Leica Microsystems) and 63× oil-immersion objective (numerical aperture 1.4). Immunofluorescent levels were quantitated using Imaris software (Bitplane). For the BRET assays HeLa cells were transfected using GeneJuice transfection reagent (EMD Millipore) with 100 ng/well of the DNA construct coding for BRET donor (RLuc-β-arrestin1 or β-arrestin2-RLuc) and increasing (100–1000 ng/well) amounts of the construct coding for BRET acceptor (human FFAR4-GFP or FFAR1-GFP, Origene). One day after transfection the cells were harvested and re-plated in 96-wells microplates, and 24 h later the media was replaced by Hanks Buffer Salt Solution and the luciferase substrate coelenterazine h (Promega) was added (5 µM) with or without DHA. Emitted luminescence and fluorescence were measured simultaneously using the Mithras^tm^ fluorescence-luminescence detector (Berthold Technologies). Cells expressing BRET donors alone were used to determine background. The BRET ratio was calculated as: (emission at 540 nm/emission at 480 nm) after addition of Coelenterazine h. The results were expressed in delta milli-BRET units (mBRET), 1 delta mBRET corresponding to the BRET ratio multiplied by 1000 for the treated condition minus BRET ratio multiplied by 1000 for control condition.

### ImageStream flow cytometry

The p65 subunit of NF-κB was visualized by indirect labeling using fixed cells. DAPI was added to visualize the nucleus prior to ImageStream Mark II (Amnis) analysis. Cell populations were hierarchically gated for single cells that were in focus and positive for both DAPI and p65. Based on the DAPI intensity histogram those cells in the G0 and G1/S phase gate were used to acquire 500–1000 GFP positive cells. Following data acquisition, the spatial relationship between the NF-κB and nuclear images was measured using the ratio translocation feature in the IDEAS software package (Amnis).

### Intracellular calcium assay

BMDMs pre-treated for 2 h with pertussis toxin (200 ng/ml), or not, were seeded at 10^5^ cells per 100 µl loading medium (RPMI 1640, 10% FBS) into poly-d-lysine coated 96-well black wall, clear-bottom microtiter plates (Nalgene Nunc). An equal volume of dye loading buffer (FLIPR Calcium 4 assay kit, Molecular Devices) in Hank's balanced salt solution supplemented with 20 mM HEPES and 2 mM probenecid was added. Cells were incubated for 1 h at 37°C before adding DHA then the calcium flux peak was measured using a FlexStation 3 (Molecular Devices). DHA was diluted in the assay loading buffer and sonicated before addition. The data was analyzed with SOFT max Pro 5.2 (Molecular Devices). Data is shown as fluorescent counts and the y-axis labeled as Lm1.

### Statistics

Data is shown as mean plus or minus one standard deviation. All results were analyzed using Prism 6 (GraphPad software) and statistical differences between datasets were calculated using unpaired t test.

## Results and Discussion

### DHA inhibits Inflammasome activation in macrophages

**Figure 1:**
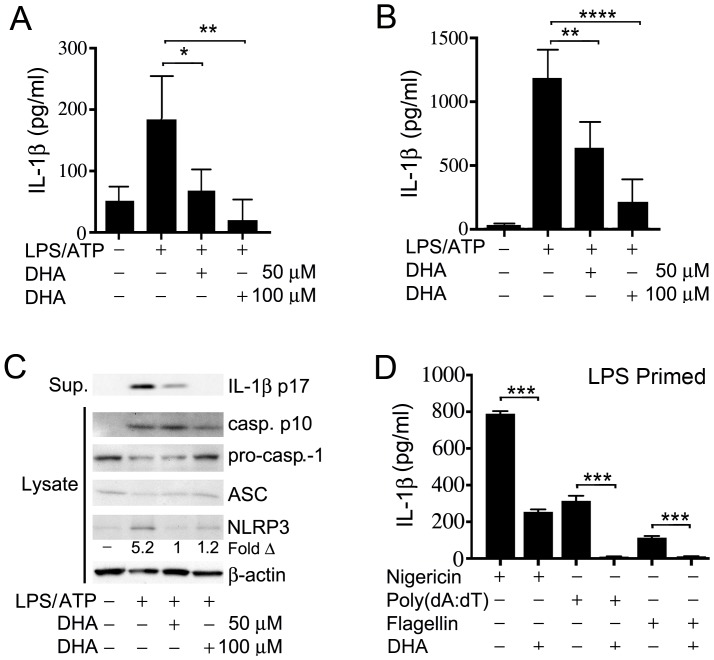
DHA suppresses inflammasome induced IL-1β production. LPS primed. (A) THP-1 cells and (B) BMDMs were stimulated with ATP. DHA (50 uM or 100 uM) added or not at the priming step. Supernatants were collected and an ELISA was performed to check IL-1β levels. (C) Western blot analysis of cell supernatants and lysates from LPS primed THP-1 cells treated with ATP in the presence or absence of DHA. The indicated samples were analyzed for mature IL-1β, pro- and mature caspase-1, ASC, NLRP3, and actin. (D) BMDMs were LPS primed and stimulated with the inflammasome inducers nigericin, Poly(dA:dT), and flagellin. DHA was added, or not, at the priming step. Supernatants were collected and IL-1β levels checked by ELISA. Results are representatives of a minimum of three independent experiments. * P<0.05, **P<0.001, ***P<0.0002, ****P<0.0001.

To test whether ω3 FFA affected IL-1β production by macrophages following exposure of the cells to a known NLRP3 activator we initially chose to treat the human macrophage cell line THP-1 with LPS and ATP in the presence or absence of DHA. LPS provides a priming signal that triggers the translocation of NF-κB from the cytosol to the nucleus of the cells [11]. This increases the expression of NF-κB responsive genes such as *NLRP3* and *IL1B*. ATP provides a second signal by binding to the cell membrane receptor P2X7, which triggers a K^+^ efflux and the assembly of the inflammasome components [4]. These experiments demonstrated that the addition of DHA at physiologically achievable concentrations resulted in a significant reduction in IL-1β secretion by the stimulated THP-1 cells (Figure 1A). Similar results were found with the related ω3 FFA EPA (data not shown). To confirm these results we also examined the effect of DHA on primary mouse BMDMs. Again DHA potently inhibited IL-1β secretion following stimulation of the cells with LPS and ATP (Figure 1B). To determine if DHA treatment affected the expression of inflammasome components pro-caspase-1, ASC and NLRP3, we immunoblotted cell lysates prepared from BMDM cell lysates from non-treated, or from LPS +ATP treated cells in the presence or absence of DHA. These results showed a marked reduction in NLRP3 protein expression in DHA treated macrophages while ASC and pro-caspase-1 levels were not significantly affected (Figure 1C). We verified inflammasome activation by immunoblotting the cell supernatants for mature IL-1β. These results indicate that DHA treatment affects NLRP3 inflammasome activity by limiting their assembly as low NLRP3 levels are known to constrain the assembly process [4].

To determine whether DHA reduced IL-1β secretion by BMDMs in response to other inflammasome activators, we used another NLRP3 inflammasome activator, nigericin, as well as activators of AIM2 and NAIP5/NLRC4 inflammasomes, double stranded DNA and flagellin, respectively. Nigericin is a K^+^ ionophore that stimulates IL-1β secretion by LPS primed mouse BMDMs. These results showed that DHA reduced nigericin induced IL-1β secretion by approximately 75% (Figure 1D). Double stranded DNA is detected by the intracytoplasmic DNA sensor AIM2, which with ASC and Caspase-1 assembles the AIM2 inflammasome. Bacterial flagellin is detected by the NAIP5/NLRC5 hetero-oligomeric inflammasome that also triggers caspase-1-dependent IL-1β secretion. In our experiments DHA potently reduced IL-1β secretion from mouse BMDMs stimulated by either Poly(dA:dT) or flagellin (Figure 1D). These results show that DHA can reduce the activity of several different types of inflammasomes.

### DHA suppresses the LPS-induced translocation of NF-κB from the cytoplasm to the nucleus in THP-1 cells and murine BMDMs

**Figure 2:**
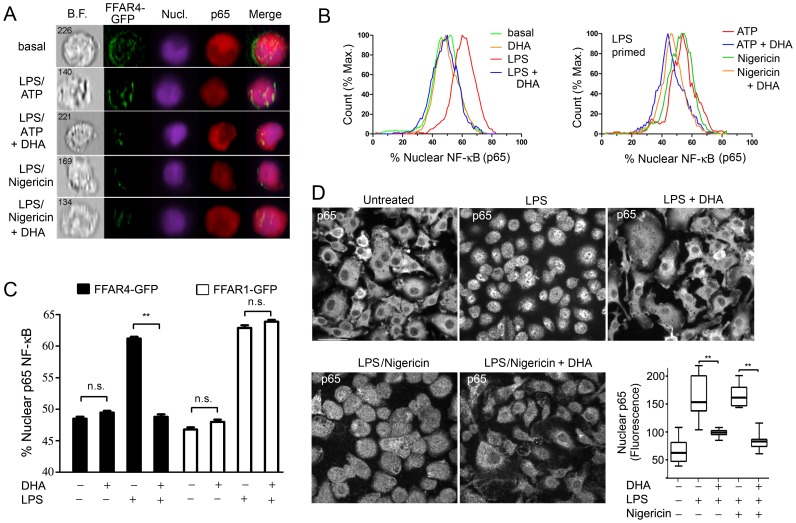
DHA inhibits the translocation of NF-κB to the nucleus. FFAR4-GFP expressing LPS primed non-differentiated THP-1 cells were stimulated with ATP or nigericin. DHA (100 µM) was added or not at the priming step. PFA fixed cells were permeablized and then stained with p65 antibody and analysis was performed using an ImageStream instrument. Shown are the (A) Imaging results and (B) Flow cytometry results. (C) ImageStream analysis of nuclear p65 NF-κB in non-differentiated THP-1 cells expressing FFAR1-GFP or FFAR4-GFP and exposed to LPS, or not, in the presence of DHA (50 µM), or not. (D) Confocal microscopy of BMDMs LPS primed and treated with nigericin in the presence, or absence of DHA, to examine the status of NF-κB translocation by p65 immunostaining. Shown are representative individual images. Scale bar is 20 µM. Whisker plot shows the amount of nuclear p65 immunofluorescence in the nuclei of BMDMs treated as indicated. Data are representative of three independent experiments. **P<0.001, n.s-not significant.

The reduced level of NLRP3 in LPS primed mouse BMDMs treated with DHA observed above; the reported inhibition of LPS induced phosphorylation of IKKβ in DHA treated RAW 264.7 cells [2]; and the known requirement for NF-κB translocation for successful LPS priming [4] prompted us to examine whether DHA affected the nuclear translocation of NF-κB. As non-differentiated THP-1 cells express low levels of *Ffar4* mRNA, we transfected them with the ω3 FFA receptor FFAR4 (GPR120) fused to GFP. We used undifferentiated cells because of their lower basal expression of nuclear p65 NF-κB. We LPS primed the transfected cells in the presence or absence of DHA and performed a flow based imaging assay to determine the amount of nuclear p65 NF-κB in the FFAR4-GFP positive cells. We did a similar experiment, but also included an inflammasome activator. These results showed that DHA potently reduced the nuclear translocation of p65 NF-κB following LPS priming or LPS priming plus nigericin (Figure 2A & B). Next, we compared the translocation of p65 NF-kB in FFAR4-GFP or FFAR1-GFP expressing undifferentiated THP-1 cells. Focusing only on the FFAR4-GFP or FFAR1-GFP positive cells, we found that FFAR4 mediated the inhibitory effect of DHA, while FFAR1 did not (Figure 2C). Next, we checked whether DHA suppressed p65 NF-κB nuclear translocation following LPS priming and inflammasome activation in mouse BMDMs. In the absence of LPS priming the majority of p65 NF-κB resided in the cytosol, while exposure to LPS or LPS plus nigericin shifted a portion of p65 NF-κB to the nucleus, but the addition of DHA largely prevented this shift (Figure 2D). Thus, DHA signaling can dampen inflammasome activation by limiting the initial priming step likely by engaging FFAR4, not FFAR1.

### Reducing FFAR4 expression limits the DHA-mediated suppression of IL-1β secretion

**Figure 3:**
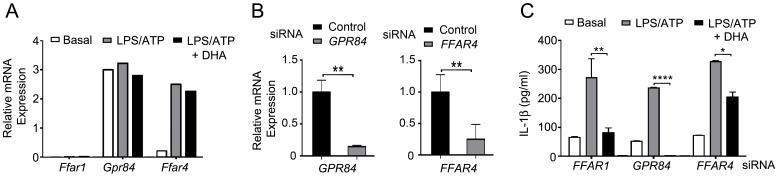
DHA-mediated suppression of inflammasome activity depends upon FFAR4. (A) Quantitative RT-PCR to detect *Ffar1*, *Gpr84*, and *Ffar4* mRNA expression in BMDMs. Cells were LPS primed and ATP treated. DHA (100 µM) added during priming step, or not. Data is relative to *Gapdh* expression x 10^5^. Experiment performed 2 times with similar results. (B) Quantitative RT-PCR to detect *GPR84* or *FFAR4* mRNA in LPS primed THP-1 cells expressing siRNAs targeting *FFAR4* or *GPR84*, respectively. The results were normalized to β-actin expression and shown as relative to control. (C) IL-1β ELISA to assess inflammasome activity in FFAR1, GPR84, or FFAR4 knock-down cells. LPS primed THP-1 cells expressing siRNAs targeting *FFAR1*, *GPR84*, or *FFAR4* were stimulated with ATP. DHA added during the priming step. Similar results from 3 experiments. *P<0.05, **P<0.001, ****P<0.0001.

Of the six G-protein coupled receptors (GPR) receptors that recognize FFAs, FFAR1, FFAR2 (GPR43), FFAR3 (GPR41), FFAR4, GPR84, and GPR119; only FFAR1 and FFAR4 have been shown to bind ω3 FFA 1 [12], [13]. *Ffar1* mRNA is detected primarily in pancreatic β-cells while *Ffar4* mRNA is found in the intestine, adipocytes, and macrophages [13]. In the mouse cell line Raw 264.7 DHA mediated its suppressive effects by engaging FFAR4 [2]. Using RT-PCR we showed that BMDMs constitutively express *Gpr84*, low levels of *Ffar4*, and almost undetectable levels of *Ffar1* mRNA (Figure 3A). A four hour exposure to LPS increased *Ffar4* mRNA expression approximately 12-fold compared to non-stimulated cells, but had little effect of *Ffar1* mRNA expression (Figure 3A). To check the involvement of FFAR4 in DHA-mediated suppression of inflammasome activation and secretion of IL-1β, we first verified that differentiated THP-1 cells expressed *FFAR4* mRNA and then reduced its expression using a siRNA pool. Controls were siRNAs directed at *GPR84* mRNA or an irrelevant target (Figure 3B). Next, we checked the impact of reducing FFAR4 on inflammasome activity. We found that the knockdown of *FFAR4* mRNA significantly reduced the suppression of IL-1β production by DHA while the *GPR84* knockdown had little effect (Figure 3C). Together these results indicate that DHA predominately uses FFAR4 to suppress NLRP3 inflammasome activity in mouse BMDMs and in a differentiated human monocyte cell line.

### DHA triggers an increase in intracellular calcium and the recruitment of β-arrestins to FFAR4, which helps suppress IL-1β production

**Figure 4:**
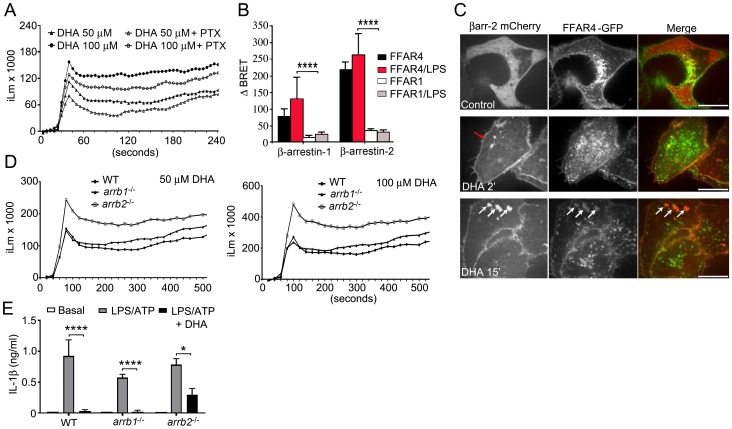
DHA elicits a calcium flux and its suppression of inflammasome activity partially depends upon β-arrestin2 not β-arrestin1. (A) DHA elicited changes in intracellular Ca^2+^ in BMDMs treated with pertussis toxin (200 ng/ml), or not, for 2 hours prior to the assay. (B) BRET analysis using HeLa cells expressing a BRET donor (RLuc-β-arrestin1 or β-arrestin2-RLuc) and increasing amounts of a BRET acceptor (FFAR4-GFP or FFAR1-GFP) stimulated with LPS, or not, prior to addition of DHA. The BRET ratios were calculated and the change in BRET determined. (C) Confocal microscopy of HeLa cells expressing FFAR4-GFP and β-arrestin-2 mCherry following exposure to DHA. Red arrow indicates β-arrestin-2 recruitment and the white arrows intracellular co-localization of β-arrestin-2 and FFAR4. Scale bar is 10 µM. (D) Intracellular calcium response to DHA of BMDMs prepared from wild type and β-arrestin knock-out mice. (E) ELISA to measure IL-1β in cell supernatants conditioned by LPS primed and ATP treated BMDMs from WT, *arrb1* ^−/−^, and *arrb2* ^−/−^ mice. DHA (100 M) was added at the priming step. Results are from 3 independent experiments. * P<0.05, ****P<0.0001.

FFAR4 has been reported to signal via the heterotrimeric G-protein Gq [2]. Engagement of Gq-linked GPRs usually leads to an increase intracellular calcium levels by the activation of phospholipase Cβ. To determine if DHA elicited a rise in intracellular calcium in mouse BMDMs, we challenged the cells, which had been pre-treated with pertussis toxin or not, with DHA. Pertussis toxin is a known inhibitor of Gi-linked receptors, which will not impact signaling through a Gq-linked receptor. Treatment of BMDMs with DHA resulted in modest, but prolonged increase in intracellular calcium, which was largely insensitive to pertussis toxin (Figure 4A).

GPRs signal not only by the activation of G-proteins, but also by the recruitment of β-arrestins, which serve as a signaling platform for the activation of other signal transducers [9]. The suppression of Toll receptor induced IL-6 and TNF-α production by engagement of FFAR4 has been shown to depend on β-arrestin-2 [2]. Following engagement of FFAR4 β-arrestin2 recruited TAB1, which interacted with TAK1. This inhibited TLR4 induced activation of both NF-κB and Jun kinase. We tested whether FFAR1 and FFAR4 recruited β-arrestin1 and/or β-arrestin2 following DHA treatment using bioluminescence resonance energy transfer (BRET) assays (Figure 4B). Following DHA treatment FFAR4 could recruit both β-arrestin1 and β-arrestin2, although a stronger change in the BRET signal occurred with β-arrestin2. In contrast, substituting FFAR1 for FFAR4 resulted in little or no DHA induced change in the BRET signal with either β-arrestin. Next, we co-transfected HeLa cells with FFAR4-GFP and β-arrestin2-mCherry, stimulated the cells with DHA or not, and imaged the cells by confocal microscopy. FFRA4 localized predominately at the cell membrane although some of the protein was likely retained in intracellular compartments. In contrast, β-arrestin2 largely resided in the cytoplasm. DHA treatment resulted in a strong shift of β-arrestin-2 from the cytoplasm to the cell membrane and a partial internalization of FFAR4, which co-localized with β-arrestin2 in the cytoplasm (Figure 4C). We found similar results when we substituted THP-1 cells for the HeLa cells (data not shown). Together these results argue that FFAR4 rather than FFAR1 is the relevant ω3 FFA receptor involved in limiting inflammation and likely inflammasome activation in mouse and human macrophages.

To determine which β-arrestin functioned to regulate the DHA responses in primary macrophages, we examined the rise in intracellular calcium following exposure of WT, *Arrb1* ^−/−^, *Arrb2* ^−/−^ BMDMs to DHA. We found that the loss of β-arrestin2, but not β-arrestin1, led to a higher intracellular calcium signal following exposure to either 50 or 100 µM DHA (Figure 4D). This result argues that in BMDMs, β-arrestin2 preferentially targets FFAR4. To assess β-arrestin involvement in the DHA-mediated inhibition of inflammasome activity, we LPS primed BMDMs from *Arrb1* ^−/−^ and *Arrb2* ^−/−^ mice and treated them with ATP in the presence or absence of DHA. The results from these experiments demonstrated that loss of β-arrestin2 partially reversed the DHA triggered suppression, while loss of β-arrestin1 had no effect (Figure 4E). These data indicate that β-arrestin2 signaling contributes to the DHA mediated reduction in inflammasome activation, but that other signaling pathways also contribute.

### DHA increases autophagosome formation in macrophages

**Figure 5:**
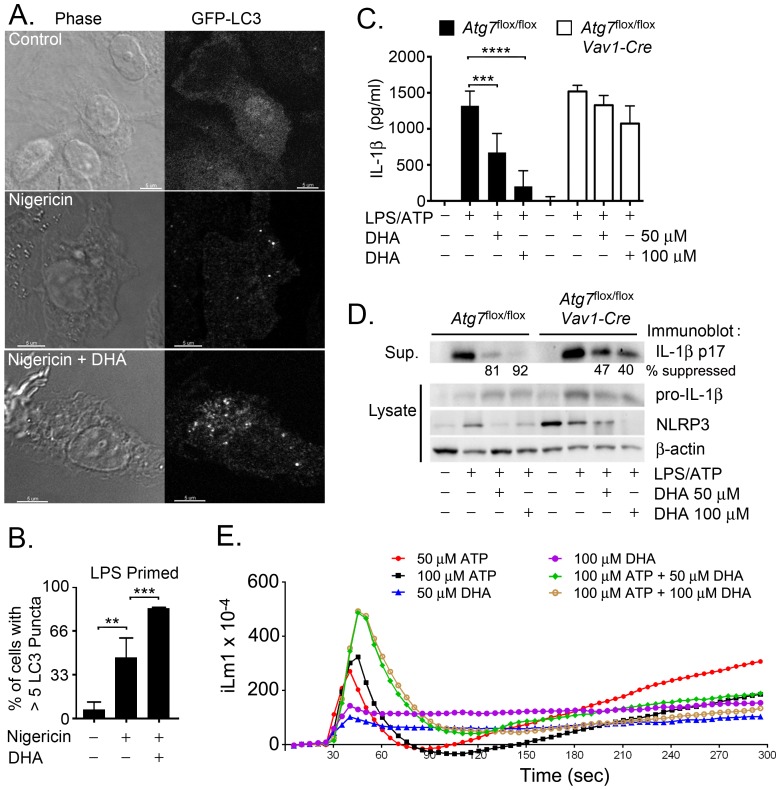
DHA regulates inflammasome activity by promoting autophagy. (A) Confocal microscopy of BMDMs from GFP-LC3 mice primed with LPS and treated with nigericin. DHA (50 µM) was added at the priming step. Scale bar is 5 µM. (B) Number of cells with >5 GFP-LC3 puncta per cell were determined for each treatment group. Data are from 3 independent experiments. (C) ELISA to measure IL-1β in supernatants conditioned by BMDMs prepared from *Atg7* ^flox/flox^ or *Atg7* ^flox/flox^ *Vav1-Cre* mice. The cells were primed with LPS and treated with ATP. DHA was added at the priming step. Results are from 3 independent experiments. (D) Western blot analysis of supernatants and cell lysates from BMDMs prepared from *Atg7* ^flox/flox^ or *Atg7* ^flox/flox^ *Vav1-Cre* mice. Cells were LPS primed and treated with ATP. DHA was added at the time of priming. The numbers indicate the relative expression of IL-1β in the cell supernatants. **P<0.001, ***P<0.0002, ****P<0.0001. (E) DHA and ATP triggered changes in intracellular Ca^+2^ in mouse BMDMs. The cells were exposed to the indicated concentrations of DHA and/or ATP. Results from one experiment performed in triplicate. Representative of three experiments performed.

A previous report from our lab demonstrated that autophagy can limit inflammasome activity by delivering inflammasomes to autophagosomes for subsequent lysosome mediated destruction [10]. Suggesting that DHA might reduce inflammasome activity by inducing autophagy several reports have shown that exposure of a variety of cell lines to ω3 FFA triggers autophagy [14], [15]. To determine whether DHA induced autophagosomes in mouse BMDMs we isolated BMDMs from GFP-LC3 mice [8] and treated them with DHA. However, DHA treatment did not increase the number of cells with GFP-LC3 puncta (data not shown). When we LPS primed and treated the cells with nigericin the number of GFP-LC3 puncta in the BMDMs did increase. Furthermore, the addition of DHA significantly enhanced the number of cells with more than 5 GFP-LC3 puncta per cell (Figure 5A & B). Similar results were found when cells were stimulated with LPS + ATP in the presence or absence of DHA (data not shown). To test whether autophagosome formation contributed to the suppressive effect of DHA on inflammasome activity we compared mice incapable of forming autophagosomes to most stimuli to wild type mice. BMDMs from *Atg7* ^flox/flox^ and *Atg7* ^flox/flox^ *Vav-1Cre* mice were stimulated with LPS + ATP in the presence of absence of DHA. The lack of ATG7 inhibited the suppressive effects of DHA on IL-1β secretion (Figure 5C). Western blot analysis of cells lysates confirmed the ELISA results (Figure 5D). The partial reduction in inflammasome activation in the ATG7 deficient macrophages is likely secondary to the effect of DHA on LPS priming while the inability of DHA to enhance autophagy may account for the sub-optimal inhibition. These results support a role for autophagy in DHA dependent suppression of inflammasome activation.

The mechanism by which DHA increases autophagosome formation is unknown although a recent study indicated that mTOR controlled autophagy required intracellular calcium signaling [16]. As both ATP and DHA are capable of eliciting an intracellular calcium flux we checked how the simultaneous addition of DHA and ATP affected the level of intracellular calcium in BMDMs. We exposed the cells to different concentrations of ATP, DHA, and to both signals together. We found that as previously described DHA triggered an increase in intracellular calcium. The addition of ATP alone elicited a higher increase in the peak intracellular calcium and a much sharper rate of increase that did DHA. The combination of ATP and DHA triggered a greater rise in intracellular calcium and a more prolonged increase than did either ATP or DHA alone. We are currently investigating whether the elevated intracellular calcium noted in these primary mouse macrophages is sufficient to trigger autophagosome formation. High concentrations of ATP have been shown to induced autophagy in human macrophages and macrophage cell lines[17].

In the course of our studies, Yan et al. published a report demonstrating that ω3 FFA suppressed macrophage NLRP3 and NLRP1b inflammasomes, but not AIM2 and NAIP5/NLRC4 inflammasomes [18]. They found roles for FFAR4, FFAR1, and β-arrestin-2 in ω3 FFA signaling. They also demonstrated a ligand induced interaction between NLRP3 and β-arrestin-2. Our results differ slightly from those of Yan et al [18]. We found a strong suppression of all the tested inflammasome activators, perhaps because we used a higher concentration of DHA and included the DHA in the priming step, thereby reducing NF-κB activation and limiting expression of some of the inflammasome components. We did not find a role for FFAR1 as we found very low levels of FFAR1 in mouse BMDMs. We did detect FFAR1 in differentiated THP-1 cells (Y. Williams-Bey, unpublished observation), but failed to find a recruitment of β-arrestin-2 to FFAR1 following exposure of cells to DHA. Our data would support a much more important role for FFAR4 than FFAR1 in inflammasome suppression. Finally we provide an additional mechanism by which ω3 FFA limit inflammasome activity and that is by enhancing autophagy

In summary, we have shown that ω3 FFA can suppress NLRP3, AIM2, and NAIP/NLCR4 inflammasome activation. We focused on NLRP3 inflammasome activation and demonstrated a requirement for FFAR4. Mouse BMDMs express *Ffar4* and LPS stimulation increased *Ffar4* mRNA levels. DHA stimulation recruited β-arrestin-1 and -2 to FFAR4 and caused receptor internalization, but only β-arrestin-2 helps mediated the suppressive effects of DHA. We identified two mechanisms by which DHA suppressed macrophage inflammasome activity, first, it impaired priming by inhibiting NF-κB activation likely via a β-arrestin-2 dependent mechanism and, second, it enhanced autophagy, thereby reducing inflammasome complex formation or presenting inflammasome components for destruction. Our studies support the further study and use of ω3 FFA in those clinical situations characterized by excessive macrophage inflammasome activity.
